# Clinical and Psychological Variables in Female Patients with Cervical Syndromes: A Cross-Sectional and Correlational Study

**DOI:** 10.3390/healthcare10122398

**Published:** 2022-11-29

**Authors:** Sara Cabanillas-Barea, Andoni Carrasco-Uribarren, Ricardo Medrano-de-la-Fuente, Sandra Jiménez-del-Barrio, Pilar Pardos-Aguilella, Silvia Pérez-Guillén, Luis Ceballos-Laita

**Affiliations:** 1 Faculty of Medicine and Health Sciences, International University of Catalonia, 08195 Sant Cugat del Vallés, Spain; 2 Department of Surgery, Ophthalmology, Otorhinolaryngology and Physiotherapy, University of Valladolid, 42004 Soria, Spain; 3 Clinical Research in Health Sciences Group, University of Valladolid, 42004 Soria, Spain; 4 Faculty of Health Sciences, University of Zaragoza, 50009 Zaragoza, Spain

**Keywords:** cervical, spine, pain, anxiety, depression, kinesophobia

## Abstract

Background: The objectives of this study were: (1) to compare the pain intensity, cervical range of motion (ROM), psychological distress and kinesiophobia in patients with cervicogenic dizziness (CGD), tension-type headache (TTH), and mechanical chronic neck pain (MCNP); and (2) to investigate the relationships between pain intensity and cervical ROM and between psychological distress and kinesiophobia. Methods: a cross-sectional and correlational study was designed. In total, 109 patients (32 patients with CGD, 33 with TTH and 44 with MCNP) were included. Pain intensity, cervical ROM, psychological distress and kinesiophobia were assessed. Results: Statistically significant differences were found between the groups in pain intensity, psychological distress and kinesiophobia. The patients with MCNP showed higher pain intensity compared to the other groups (*p* < 0.001). The patients with CGD showed higher depression and kinesiophobia values compared to the MCNP and TTH groups (*p* < 0.05). No differences were found for cervical flexion, extension, lateral flexion, or rotation ROM (*p* > 0.05). The CGD and MCNP groups found a moderate positive correlation between psychological distress and kinesiophobia (*p* < 0.05). The patients with TTH and MCNP showed a moderate positive correlation between pain intensity, psychological distress and kinesiophobia (*p* < 0.05). Conclusion: Pain intensity, psychological distress and kinesiophobia should be considered in the three groups. Psychological distress was correlated with kinesiophobia in the CGD and MCNP groups. The MCNP group showed a correlation between pain intensity, psychological distress and kinesiophobia.

## 1. Introduction

Symptoms related to the cranial and neck areas are among the most prevalent reasons for consultation in primary care and they are important causes of disability in the United States, creating significant socioeconomic costs [1,2,3]. The Global Burden of Disease study from 2019) reported a prevalence of 223 million people suffering neck pain [4]. Furthermore, in the most recent study, neck pain ranked 19th, as measured by disability-adjusted life years for ages 25 to 49 [4]. The incidence of pain in these regions is increasing [5]. Cranio-cervical pain (CP) prevalence increases with age and is more common in women in their fifth decade [6].

The causes of CP are diverse, which makes it challenging for clinicians to locate the source of the symptoms [7]. The symptoms and signs of CP may be related to a feeling of instability, headache, stiffness and, sometimes, kinesiophobia, depression and anxiety [8,9,10,11]. These symptoms and signs present during the assessment are important findings that allow clinicians to classify patients, decide on the objectives of the intervention and offer prognosis [5].

In clinical practice, patients usually complain about dizziness, headache and mechanical pain [5,12,13]. Depending on the type of symptoms and other signs, such as restrictions on the range of movement (ROM), the most common classifications in clinical care are cervicogenic dizziness (CGD), tension-type headache (TTH) and mechanical chronic neck pain (MCNP) [5,13,14]. These clinical symptoms are more frequent in women than in men [13,15,16].

The clinical characteristics are different in each group. The primary symptoms of patients diagnosed with CGD are dizziness and neck pain; in patients diagnosed with TTH, the main symptoms are headaches and neck pain; and patients diagnosed with MCNP solely experience neck pain. To assess the symptoms, clinicians usually use valid and reliable instruments to measure these outcome variables, such as the visual analogue scale (VAS) and goniometers or inclinometers. The VAS is a low-cost and easy-to-use tool for patients with CP in clinical practice [17]. Goniometers measure ROM in clinical care and in scientific terms [18].

However, the increasing evidence about the relationship between CP prognosis and other psychosocial factors [11,19] has encouraged clinicians to record new variables in their routine practice. These variables include psychological distress (anxiety and depression) and fear of movement, or kinesiophobia. Self-report questionnaires have become popular and have been proven to be valid and reliable tools [20]. It is unknown whether there are differences between the aforementioned groups of patients with regards to psychological distress and kinesiophobia. A knowledge of whether psychological distress and kinesiophobia are present in the previously defined groups and a comparison of these characteristics between the groups could help clinicians to improve the wellbeing of patients. It has been observed that pain education can provide benefits for the treatment of CP [21]; if there are differences between groups, the treatment and education provided can be emphasized, based on the differences found.

Some authors suggest that psychosocial factors are frequent findings in patients with CP. Some of these variables may be predicted in patients with CP based on pain intensity. For this reason, it is suggested that psychological distress and kinesiophobia may be pain mediators in patients with CP [16,22,23,24,25]. Recent evidence suggests a relationship between pain intensity, cervical ROM, psychological distress and kinesiophobia [16,22,23,24,25]. However, there is a lack of evidence comparing pain intensity, cervical ROM, psychological distress and kinesiophobia between female patients with CGD, TTH and MCNP. In addition, it is still unclear whether a correlation between pain intensity, ROM, psychological distress and kinesiophobia exists in these different types of patients. Therefore, this study presents two objectives: (1) to compare the pain intensity, cervical ROM, psychological distress and kinesiophobia in female patients with CGD, TTH and MCNP; and (2) to investigate the relationships between pain intensity and cervical ROM and between psychological distress and kinesiophobia in females.

## 2. Materials and Methods

### 2.1. Study Design

This cross-sectional and correlational study was performed between February 2019 and March 2021. The Research Ethics Committee of Aragón (PI15/229) approved the study protocol, which followed the Strengthening the Reporting and Observational Studies in Epidemiology (STROBE) guidelines [26]. According to the Declaration of Helsinki (2013), all the participants who agreed to participate signed the informed-consent form.

### 2.2. Participants

One hundred and nine female participants were included in the study (32 diagnosed with CGD, 33 diagnosed with TTH and 44 diagnosed with MCNP). The inclusion criteria in the CGD group were dizziness, neck pain and age > 18 years. The inclusion criteria in the TTH were: TTH diagnosis according to the ICHD-III [13] and aged > 18 years. The inclusion criteria in the MCNP group were pain in the neck region for at least 3 months age > 18 years. Exclusion criteria were the same for the three groups: male patients; history of cranial or neck trauma, cervical radiculopathy, or acute cranial or neck pain; previous surgery in the cranial, neck or shoulder region; deformities, infections or malignancy; previous physiotherapy treatments in the last 3 months; other general red flags or contraindications (such as positive Klein’s test, or hyperlipidemia).

### 2.3. Procedure

Before the enrolment, all the participants were diagnosed with CGD, TTH or MCNP by medical doctors from primary-care centers in Delicias Norte and Delicias Sur, Zaragoza (Spain).

**Figure 1:**
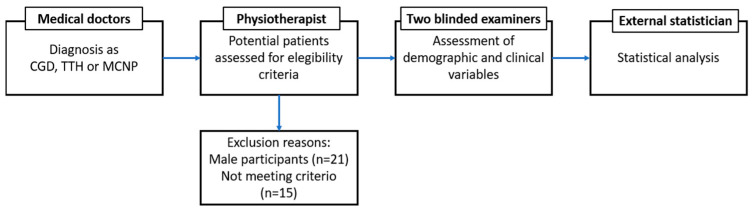
Procedure: Research timeline.

Demographic variables (sex, age, weight, height) were assessed for descriptive purposes. The outcome variables considered in this study were pain intensity, cervical ROM, psychological distress and kinesiophobia. Two blinded examiners who did not know the assigned group measured all the outcome variables. The procedure process is shown in the Figure 1.

### 2.4. Outcome Variables

#### 2.4.1. Pain Intensity

Pain intensity was measured using the 100-mm Visual Analog Scale (VAS), in which 0 represented no pain and 100 represented the most intense pain imaginable. The VAS has been shown to be a valid and reliable instrument for the measurement of pain in patients with CP [17].

#### 2.4.2. Cervical ROM

Active cervical flexion, extension, lateral flexion and rotation ROM were registered using the Cervical Range of Motion Instrument (CROM, Performance Attainment Associates, St. Paul, MN, USA). The patients were placed with the cervical spine in a neutral position. The examiner recorded the initial angle in the selected plane of the CROM. Next, the patients were asked to perform flexion, extension, lateral flexion, or rotation without pain and avoiding trunk or shoulder movement. The examiner ensured the correct running of the test. This protocol was used for all movements and repeated three times for each [27]. The test-retest–reliability of this protocol has shown an Intraclass Correlation Coefficient (ICC) between 0.86 and 0.96 [28].

#### 2.4.3. Psychological Distress

Psychological distress was assessed using the Spanish version of the Hospital Anxiety and Depression Scale (HADS). The HADS questionnaire includes 14 items to assess anxiety and depression symptoms. Each item is rated from 0 (no distress) to 3 (maximum distress). The cut-off score for the presence of psychological distress is >8 points. The cut-off scores for the anxiety and depression subscales are as follows: normal (0 to 7 points), borderline abnormal (8 to 10 points) and abnormal (>11 points) [29]. The sensitivity and specificity of this cut-off is 0.80 [30].

#### 2.4.4. Kinesiophobia

Kinesiophobia was registered with the Spanish version of the TKS-11 questionnaire. TSK-11 includes 11 items to assess fear of movement. Each item was rated from 1 (strongly disagree) to 4 (strongly agree). The test–retest reliability of this questionnaire has shown an ICC of 0.95 [31].

### 2.5. Statistical Analysis

Statistical Package for the Social Sciences (SPSS) software version 20.0 for Windows was used for statistical analysis [32]. Continuous variables were presented as mean (M) and standard deviation (SD). Categorical variables were presented as frequency and percentages. The variables’ normal or non–normal distribution was calculated using the Shapiro–Wilk test. Between-group comparisons of clinical and sociodemographic data were analyzed using the one-factor analysis of variance (ANOVA). When a statistically significant difference was noted, the Bonferroni post hoc was analyzed to study comparisons between each group. Spearman Rho was used due to the small number of participants. A p-value < 0.05 was considered statistically significant. The rank correlation coefficients were interpreted as weak (rho = 0–0.3), moderate (rho = 0.3–0.5), strong (rho = 0.5–0.7) or very strong (rho = 0.7–1) [33].

## 3. Results

One-hundred and forty-five participants were recruited for the study. Thirty-six were excluded for not meeting the three groups’ eligibility criteria. One-hundred and nine female patients with CGD, TTH or MCNP were finally included in the study. The demographic variables are shown in Table 1.

The analysis of the variance (ANOVA) showed statistically significant differences between the three groups for pain intensity and psychological distress (*p* < 0.05) (Table 2). The MCNP group showed higher pain intensity compared to the CGD group (Δ21.66; 95%CI: 12.35, 30.96; *p* < 0.001) and to the TTH group (Δ27.34; 95%CI: 18.89, 35.80; *p* < 0.001). The CGD group showed higher values of psychological distress (Δ3.15; 95%CI: 0.47, 5.84; *p* = 0.022) and depression (Δ2.58; 95%CI: 1.29, 3.87; *p* < 0.001) compared to the MCNP group and higher values of kinesiophobia compared to the TTH group (Δ3.27; 95%CI: 0.28, 6.28; *p* = 0.032).

Concerning the psychological-distress cut-offs, 84.37% of the patients in the CGD group, 63.63% in the TTH group and 68.12% in the MCNP group presented symptoms of psychological distress. The frequencies of the HADS total scale and anxiety and depression subscales are presented in Table 3.

The correlation analysis showed a moderate positive correlation between psychological distress and kinesiophobia in the CGD group (r = 0.429; *p* = 0.014) and the MCNP group (r = 0.424; *p* = 0.004).

Additionally, associated with pain intensity, a moderate positive correlation was found with psychological distress and kinesiophobia, albeit only in the MCNP group (Table 4).

The statistical power was calculated with G*Power version 3.1 (https://www.psychologie.hhu.de/arbeitsgruppen/allgemeine-psychologie-und-arbeitspsychologie/gpower, accessed on 17 October 2022)with a 95% confidence interval and a sample of 109 participants showed estimated differences between the groups of 87.2% to 99% for pain intensity, psychological distress and kinesiophobia. The statistical power obtained for the correlation was 99% for psychological distress and kinesiophobia and for pain, psychological distress and kinesiophobia correlation.

## 4. Discussion

The aims of this study were: (1) to compare the pain intensity, ROM, psychological distress and kinesiophobia in female patients with CGD, TTH and MCNP in females; and (2) to investigate the relationships between psychological distress and kinesiophobia and between pain intensity and cervical ROM in females. Concerning the first objective, the results of the present study showed that the patients with MCNP presented higher pain-intensity values than those with CGD and TTH. The patients with CGD presented higher values of psychological distress and depression compared to the patients with MCNP and higher values of kinesiophobia compared to the patients with TTH and MCNP. Regarding the second objective, the CGD and MCNP groups demonstrated a moderate positive correlation between psychological distress and kinesiophobia. The MCNP group presented a positive moderate correlation between pain intensity, psychological distress and kinesiophobia.

Statistically significant differences were found in pain intensity, depression and kinesiophobia. The mean values displayed for each group in pain intensity were similar to the values reported in other studies that included the same populations [34,35]. The MCNP group presented the highest values in pain intensity; these subjects focused their attention on the intensity of their self-perceived pain. This contrasted with the subjects with TTH, who focused their attention on the intensity of their headache, as well as with the patients with CGD, who focused their attention on the self-perceived intensity of their dizziness. The mean values reported for each group for depression and kinesiophobia were similar to the results found in other studies [36,37,38,39]. The CGD group presented the highest values for depression and kinesiophobia compared to the other populations. Several studies have found that patients with CGD associated with non-specific CP often maintain maladaptive beliefs, such as fear avoidance and high levels of anxiety, depression and high disability due to dizziness [40,41,42].

No statistically significant differences were found in any group’s three planes of ROM. Cervical ROM restriction is common in most patients with CP [5,43,44]. Perhaps, for this reason, no between-group differences were found. This is one of the reasons why cervical ROM is not clinically used as a diagnostic criterion [5,45,46].

According to the cut-off points proposed by Zigmond et al. [30], more than 60% of the patients in the three groups presented psychological distress. The group-specific values were 84.37% of the patients included in the CGD group, 63.3% of those in the TTH group and 68.12% of those in the MCNP group. These results are similar to those of other studies. Corrêa-Rangel et al. [47] showed similar results to our study, with 62.7% of their patients presenting anxiety and 57.4% depression in a sample with TTH. The cross-sectional study developed by Elbinoune et al. [48] observed the prevalence of anxiety and depression in MCNP, obtaining similar results to our study, according to which 68.4% of the sample presented anxiety and 55.7% depression. There is a lack of evidence evaluating the presence of psychological distress in CGD patients, but the mean values achieved in this study were similar to those in previous studies [37,38,49].

The three groups showed a positive moderate correlation between psychological distress and kinesiophobia. This means that higher values of psychological distress were related to higher values of kinesiophobia and vice versa. It is important to note that the correlations were found in the three groups, which means that the correlations were independent of the type of diagnosis received by the patient. To the best of our knowledge, this is the first study to investigate the relationship between psychological distress and kinesiophobia in patients with CP. Therefore, these results are not comparable to those of similar studies.

In addition, the TTH group and the MCNP group showed a positive moderate correlation between pain intensity, psychological distress and kinesiophobia. However, the CGD group did not show a correlation. This means that higher pain intensity values are related to higher values of psychological distress and kinesiophobia in patients with TTH and MCNP. Pain is the most common reason for seeking medical attention in patients with TTH and MCNP [5,13]. Furthermore, several studies have shown a relationship between pain intensity and psychological distress or kinesiophobia [19,40,50]. However, patients with CGD present dizziness as the most important symptom [35]. Nevertheless, pain is also a common symptom; this fact may explain the lack of correlation between pain intensity and psychological distress or kinesiophobia.

The prevalence of CP has increased in recent years in Spain. The prevalence and intensity of chronic pain are higher among women. In addition, it has been observed that women use more medication for symptoms of pain, anxiety and depression than men [15]. Several studies concluded that the addition of pain education to manual therapy can be a very useful intervention in patients with CP [21]. From a clinical point of view, the differences between groups in pain intensity, psychological distress and kinesiophobia may help to establish new clinical criteria for the diagnosis and treatment of CGD, TTH and MCNP. However, these should be in agreement with the criteria described by the most recent clinical guidelines. In addition, the relationships found between pain intensity, psychological distress and kinesiophobia may contribute to assessing patients’ clinical prognosis.

This study has some limitations that should be described. First, since only three groups were included, the results cannot be extrapolated to other subgroups with cervical or cranial disorders. Second, the study took into account only women and the results were compared with those of other studies that did not differentiate groups of patients by sex; therefore, the patients in this study may not have been representative of the population. Third, of the pain conditions that may have influenced the status of the patients (i.e., fibromyalgia), only pain intensity was recorded. Finally, the study design allowed us to establish a correlation but not a cause–effect association for the differences found.

The present study was conducted on females. Future studies should investigate differences between genders and ages in the three groups. In addition, the design of prospective or retrospective studies may allow to describe casual associations.

## 5. Conclusions

Pain intensity, psychological distress and kinesiophobia are parameters to consider in patients with CD, TTH and MCNP. Psychological distress and kinesiophobia were shown to be positively correlated in the three groups. Pain intensity was shown to be correlated with psychological distress and kinesiophobia in the TTH and MCNP groups.

## Figures and Tables

**Table 1:** Demographic variables of the three groups.

	CGD Group M (SD) (n = 32)	TTH Group M (SD) (n = 33)	MCNP Group M (SD) (n = 44)	*p*-Value G1–G2 G1–G3 G2–G3
Age (years)	41.50 (12.12)	40.33 (14.03)	46.75 (14.75)	0.721 10.104 0.057
Weight (kg)	63.59 (10.28)	63.72 (11.94)	64.95 (12.74)	0.962 0.620 0.668
Height (cm)	160.00 (5.61)	163.70 (5.62)	162.05 (5.98)	0.052 0.135 0.225
BMI (kg/cm^2^)	24.88 (4.10)	23.35 (4.34)	24.63 (4.33)	0.149 0.800 0.204

CGD: cervicogenic dizziness; TTH: tension-type headache; MCNP: mechanical chronic neck pain; M: mean; SD: standard deviation; BMI: body-mass index.

**Table 2:** Outcome-variable comparison between the three groups.

	CGD Group M (SD)	TTH Group M (SD)	MCNP Group M (SD)	*p*-Value G1–G2–G3	*p*-Value G1–G2 G1–G3 G2–G3
Pain intensity	28.65 (22.00)	22.97 (18.27)	50.31 (18.53)	F = 21.32 <0.001	0.262 <0.001 <0.001
Flexion ROM	43.03 (10.70)	46.30 (11.43)	48.38 (8.51)	F = 2.47 0.089	0.238 0.021 0.375
Extension ROM	51.49 (11.27)	52.69 (12.65)	54.23 (13.38)	F = 0.44 0.640	0.689 0.352 0.609
Right-lateral-flexion ROM	29.31 (7.55)	32.12 (7.50)	32.79 (8.90)	F = 1.82 0.167	0.138 0.977 0.718
Left-lateral-flexion ROM	31.36 (9.36)	34.63 (7.47)	31.50 (9.19)	F = 1.52 0.222	0.124 0.949 0.114
Right-rotation ROM	57.17 (9.08)	57.30 (7.18)	58.43 (10.30)	F = 0.22 0.797	0.951 0.582 0.572
Left-rotation ROM	53.66 (10.68)	57.96 (7.30)	58.84 (10.30)	F = 2.32 0.103	0.062 0.065 0.705
HADS	13.75 (6.28)	11.33 (7.48)	10.59 (5.40)	F = 2.39 0.096	0.164 0.022 0.631
HADS anxiety subscale	7.93 (3.68)	7.06 (4.33)	7.36 (3.66)	F = 0.42 0.652	0.383 0.503 0.747
HADS depression subscale	5.81 (3.23)	4.27 (3.97)	3.22 (2.39)	F = 6.10 0.003	0.092 <0.001 0.186
TSK-11	23.22 (6.70)	19.94 (5.30)	22.77 (6.76)	F = 2.65 0.075	0.032 0.776 0.043

CGD: cervicogenic dizziness; TTH: tension-type headache; MCNP: mechanical chronic neck pain; M: mean; SD: standard deviation; ROM: range of motion; HADS: hospital anxiety and depression scale; TSK-11: Tampa scale for kinesiophobia.

**Table 3:** Frequency of psychological distress in the three groups.

	CGD Group	TTH Group	MCNP Group	*p*-Value
HADS (A/N)	27/5	21/12	30/14	0.146
HADS anxiety (A/BA/N)	8/9/15	6/4/23	9/8/27	0.402
HADS depression (A/BA/N)	3/7/22	2/3/28	0/2/42	0.032

CGD: cervicogenic dizziness: TTH: tension-type headache; MCNP: mechanical chronic neck pain; HADS: hospital anxiety and depression subscale; A: abnormal; BA: borderline abnormal; N: normal.

**Table 4:** Correlation analysis.

	HADS	TSK-11
Pain intensity		
CGD group	r = 0.061; *p* = 0.742	r = 0.037; *p* = 0.843
TTH group	r = 0.296; *p* = 0.094	r = 0.280; *p* = 0.115
MCNP group	r = 0.331; *p* = 0.028	r = 0.315; *p* = 0.037

HADS: hospital anxiety and depression scale; TSK-11: Tampa scale for kinesiophobia; CGD: cervicogenic dizziness; TTH: tensional-type headache; MCNP: mechanical chronic neck pain.

## Data Availability

The data analyzed in this study are included in this published article. The dataset is available from the corresponding author on reasonable request.
